# Overview of MicroRNA Biogenesis, Mechanisms of Actions, and Circulation

**DOI:** 10.3389/fendo.2018.00402

**Published:** 2018-08-03

**Authors:** Jacob O'Brien, Heyam Hayder, Yara Zayed, Chun Peng

**Affiliations:** Department of Biology, York University, Toronto, ON, Canada

**Keywords:** microRNA biogenesis, gene regulation, microRNA dynamics, extracellular microRNA, cell-cell communication

## Abstract

MicroRNAs (miRNAs) are a class of non-coding RNAs that play important roles in regulating gene expression. The majority of miRNAs are transcribed from DNA sequences into primary miRNAs and processed into precursor miRNAs, and finally mature miRNAs. In most cases, miRNAs interact with the 3′ untranslated region (3′ UTR) of target mRNAs to induce mRNA degradation and translational repression. However, interaction of miRNAs with other regions, including the 5′ UTR, coding sequence, and gene promoters, have also been reported. Under certain conditions, miRNAs can also activate translation or regulate transcription. The interaction of miRNAs with their target genes is dynamic and dependent on many factors, such as subcellular location of miRNAs, the abundancy of miRNAs and target mRNAs, and the affinity of miRNA-mRNA interactions. miRNAs can be secreted into extracellular fluids and transported to target cells via vesicles, such as exosomes, or by binding to proteins, including Argonautes. Extracellular miRNAs function as chemical messengers to mediate cell-cell communication. In this review, we provide an update on canonical and non-canonical miRNA biogenesis pathways and various mechanisms underlying miRNA-mediated gene regulations. We also summarize the current knowledge of the dynamics of miRNA action and of the secretion, transfer, and uptake of extracellular miRNAs.

## Introduction

The discovery of the first microRNA (miRNA), *lin-4*, in 1993 by the Ambros and Ruvkun groups in *Caenorhabditis elegans* (1, 2) has revolutionized the field of molecular biology. Years before, *lin-4* was characterized by the Horvitz's lab as one of the genes that regulate temporal development of *C. elegans* larvae (3, 4). Later in 1987, the same group found that a mutation in *lin-4* had an opposite phenotype to a mutation in another gene, *lin-14*, yet a *lin-14* suppressor mutation in a *null-lin-4* line was wildtype (5, 6). Both Ambros and Ruvkun continued to study *lin-4* and *lin-14* after leaving the Horvitz's lab, only to discover later that *lin-4* was not a protein-coding RNA but indeed a small non-coding RNA (7, 8). They also found that *lin-14* was post-transcriptionally downregulated through its 3′ untranslated region (UTR) and that *lin-4* had a complementary sequence to that of the 3′ UTR of *lin-14* (1). Therefore, they proposed that *lin-4* regulates *lin-14* at the post-transcriptional level (2). Since then, miRNAs have been detected in all animal model systems and some were shown to be highly conserved across species (9–12). New miRNAs are still being discovered (13) and their roles in gene regulation are well recognized.

miRNAs are small non-coding RNAs, with an average 22 nucleotides in length. Most miRNAs are transcribed from DNA sequences into primary miRNAs (pri-miRNAs) and processed into precursor miRNAs (pre-miRNAs) and mature miRNAs. In most cases, miRNAs interact with the 3′ UTR of target mRNAs to suppress expression (14). However, interaction of miRNAs with other regions, including the 5′ UTR, coding sequence, and gene promoters, have also been reported (15). Furthermore, miRNAs have been shown to activate gene expression under certain conditions (16). Recent studies have suggested that miRNAs are shuttled between different subcellular compartments to control the rate of translation, and even transcription (17).

miRNAs are critical for normal animal development and are involved in a variety of biological processes (18). Aberrant expression of miRNAs is associated with many human diseases (19, 20). In addition, miRNAs are secreted into extracellular fluids. Extracellular miRNAs have been widely reported as potential biomarkers for a variety of diseases and they also serve as signaling molecules to mediate cell-cell communications (21–23). In this review, we have provided a brief overview of the different pathways of miRNA biogenesis in animals and the expanding complexity of their regulation of gene expression. Moreover, we have discussed the dynamics of miRNA intracellular localization and function. Finally, we have summarized the secretion and circulation of miRNAs and the potential roles of extracellular miRNAs in mediating intercellular communications.

## Biogenesis of miRNAs

**Figure 1:**
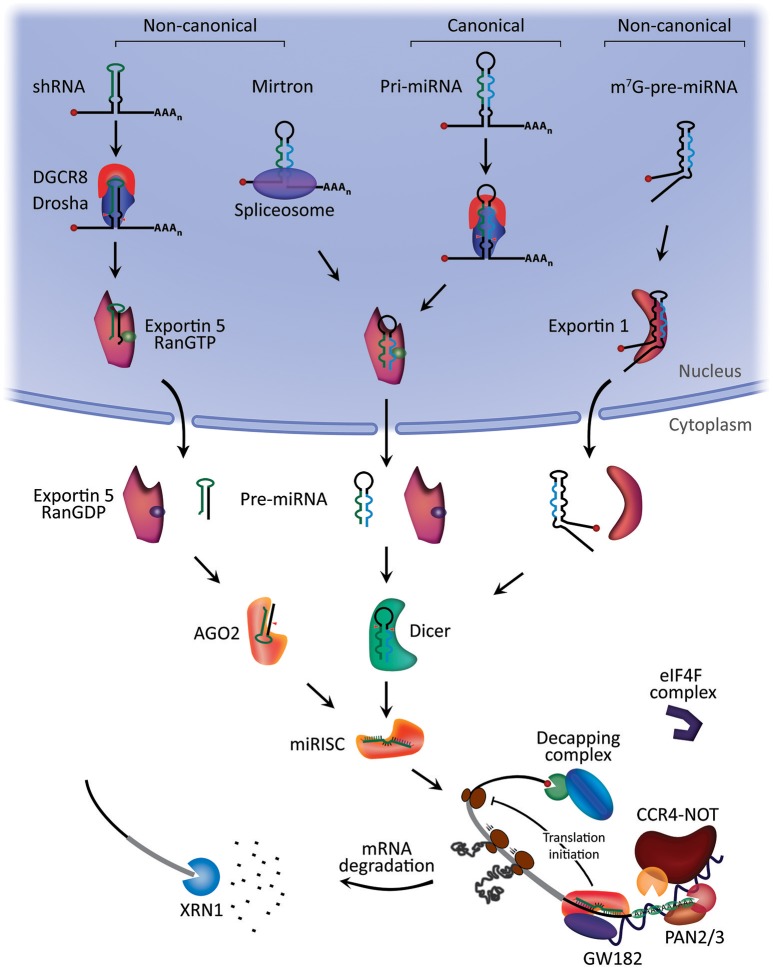
MicroRNA biogenesis and mechanism of action. Canonical miRNA biogenesis begins with the generation of the pri-miRNA transcript. The microprocessor complex, comprised of Drosha and DiGeorge Syndrome Critical Region 8 (DGCR8), cleaves the pri-miRNA to produce the precursor-miRNA (pre-miRNA). The pre-miRNA is exported to the cytoplasm in an Exportin5/RanGTP-dependent manner and processed to produce the mature miRNA duplex. Finally, either the 5p or 3p strands of the mature miRNA duplex is loaded into the Argonaute (AGO) family of proteins to form a miRNA-induced silencing complex (miRISC). In the non-canonical pathways, small hairpin RNA (shRNA) are initially cleaved by the microprocessor complex and exported to the cytoplasm via Exportin5/RanGTP. They are further processed via AGO2-dependent, but Dicer-independent, cleavage. Mirtrons and 7-methylguanine capped (m^7^G)-pre-miRNA are dependent on Dicer to complete their cytoplasmic maturation, but they differ in their nucleocytoplasmic shuttling. Mirtrons are exported via Exportin5/RanGTP while m^7^G-pre-miRNA are exported via Exportin1. All pathways ultimately lead to a functional miRISC complex. In most cases, miRISC binds to target mRNAs to induce translational inhibition, most likely by interfering with the eIF4F complex. Next, GW182 family proteins bound to Argonaute recruit the poly(A)-deadenylases PAN2/3 and CCR4-NOT. PAN2/3 initiates deadenylation while the CCR4-NOT complex completes the process, leading to removal of the m^7^G cap on target mRNA by the decapping complex. Decapped mRNA may then undergo 5′−3′ degradation via the exoribonuclease XRN1. Modified from Hayder et al. (26).

miRNA biogenesis starts with the processing of RNA polymerase II/III transcripts post- or co-transcriptionally (14). About half of all currently identified miRNAs are intragenic and processed mostly from introns and relatively few exons of protein coding genes, while the remaining are intergenic, transcribed independently of a host gene and regulated by their own promoters (13, 24). Sometimes miRNAs are transcribed as one long transcript called clusters, which may have similar seed regions, and in which case they are considered a family (25). The biogenesis of miRNA is classified into canonical and non-canonical pathways (Figure 1).

### The canonical pathway of miRNA biogenesis

The canonical biogenesis pathway is the dominant pathway by which miRNAs are processed. In this pathway, pri-miRNAs are transcribed from their genes and then processed into pre-miRNAs by the microprocessor complex, consisting of an RNA binding protein DiGeorge Syndrome Critical Region 8 (DGCR8) and a ribonuclease III enzyme, Drosha (27). DGCR8 recognizes an N6-methyladenylated GGAC and other motifs within the pri-miRNA (28), while Drosha cleaves the pri-miRNA duplex at the base of the characteristic hairpin structure of pri-miRNA. This results in the formation of a 2 nt 3′ overhang on pre-miRNA (29). Once pre-miRNAs are generated, they are exported to the cytoplasm by an exportin 5 (XPO5)/RanGTP complex and then processed by the RNase III endonuclease Dicer (27, 30). This processing involves the removal of the terminal loop, resulting in a mature miRNA duplex (31). The directionality of the miRNA strand determines the name of the mature miRNA form. The 5p strand arises from the 5′ end of the pre-miRNA hairpin while the 3p strand originates from the 3′ end. Both strands derived from the mature miRNA duplex can be loaded into the Argonaute (AGO) family of proteins (AGO1-4 in humans) in an ATP-dependent manner (32). For any given miRNA, the proportion of AGO-loaded 5p or 3p strand varies greatly depending on the cell type or cellular environment, ranging from near equal proportions to predominantly one or the other (33). The selection of the 5p or 3p strand is based in part on the thermodynamic stability at the 5′ ends of the miRNA duplex or a 5′ U at nucleotide position 1 (34). Generally, the strand with lower 5′ stability or 5′ uracil is preferentially loaded into AGO, and is deemed the guide strand. The unloaded strand is called the passenger strand, which will be unwound from the guide strand through various mechanisms based on the degree of complementarity. The passenger strands of miRNA that contain no mismatches are cleaved by AGO2 and degraded by cellular machinery which can produce a strong strand bias. Otherwise, miRNA duplexes with central mismatches or non-AGO2 loaded miRNA are passively unwound and degraded (14).

### Non-canonical miRNA biogenesis pathways

To date, multiple non-canonical miRNA biogenesis pathways have been elucidated (Figure 1). These pathways make use of different combinations of the proteins involved in the canonical pathway, mainly Drosha, Dicer, exportin 5, and AGO2. In general, the non-canonical miRNA biogenesis can be grouped into Drosha/DGCR8-independent and Dicer-independent pathways. Pre-miRNAs produced by the Drosha/DGCR8-independent pathway resemble Dicer substrates. An example of such pre-miRNAs is mirtrons, which are produced from the introns of mRNA during splicing (35, 36). Another example is the 7-methylguanosine (m^7^G)-capped pre-miRNA. These nascent RNAs are directly exported to the cytoplasm through exportin 1 without the need for Drosha cleavage. There is a strong 3p strand bias most likely due to the m^7^G cap preventing 5p strand loading into Argonaute (37). On the other hand, Dicer-independent miRNAs are processed by Drosha from endogenous short hairpin RNA (shRNA) transcripts (38). These pre-miRNAs require AGO2 to complete their maturation within the cytoplasm because they are of insufficient length to be Dicer-substrates (38). This in turn promotes loading of the entire pre-miRNA into AGO2 and AGO2-dependent slicing of the 3p strand. The 3′-5′ trimming of the 5p strand completes their maturation (39).

## Mechanisms of miRNA-mediated gene regulation

Most studies to date have shown that miRNAs bind to a specific sequence at the 3′ UTR of their target mRNAs to induce translational repression and mRNA deadenylation and decapping (40, 41). miRNA binding sites have also been detected in other mRNA regions including the 5′ UTR and coding sequence, as well as within promoter regions (42). The binding of miRNAs to 5′ UTR and coding regions have silencing effects on gene expression (43, 44) while miRNA interaction with promoter region has been reported to induce transcription (45). However, more studies are required to fully understand the functional significance of such mode of interaction.

### MicroRNA-mediated gene silencing via miRISC

The minimal miRNA-induced silencing complex (miRISC) consists of the guide strand and AGO (46). The target specificity of miRISC is due to its interaction with complementary sequences on target mRNA, called miRNA response elements (MREs). The degree of MRE complementarity determines whether there is AGO2-dependent slicing of target mRNA or miRISC-mediated translational inhibition and target mRNA decay (47). A fully complementary miRNA:MRE interaction induces AGO2 endonuclease activity and targets mRNA cleavage (47). However, this interaction destabilizes the association between AGO and the 3′ end of the miRNA promoting its degradation (48, 49).

In animal cells, the majority of miRNA:MRE interactions are not fully complementary (50). Most MREs contain at least central mismatches to their guide miRNA, preventing AGO2 endonuclease activity. Consequently, AGO2 acts as a mediator of RNA interference, similar to the non-endonucleolytic AGO family members (AGO1, 3, and 4 in humans). In many cases, a functional miRNA:MRE interaction occurs via the 5' seed region (nucleotides 2–8) (42, 51). However, additional paring at the 3′ end aids in the stability and specificity of the miRNA-target interaction (15).

The formation of a silencing miRISC complex starts with the recruitment of the GW182 family of proteins by miRISC; GW182 provides the scaffolding needed to recruit other effector proteins (52), such as the poly(A)-deadenylase complexes PAN2-PAN3 and CCR4-NOT, following miRNA:target mRNA interaction (50, 53). Target mRNA poly(A)-deadenylation is initiated by PAN2/3 and completed by the CCR4-NOT complex. The interaction between the tryptophan (W)-repeats of GW182 and poly(A)-binding protein C (PABPC) promotes efficient deadenylation (50). Subsequently, decapping takes place facilitated by decapping protein 2 (DCP2) and associated proteins (52), followed by 5′−3′ degradation by exoribonuclease 1 (XRN1) (54) (Figure 1).

### MicroRNA-mediated translational activation

Although most studies are focused on how miRNAs inhibit gene expression, some have also reported up-regulation of gene expression by miRNAs. In serum starved cells, AGO2 and another protein related to the miRNA-protein complex (microRNPs), Fragile-x-mental retardation related protein 1 (FXR1), were associated with AU-rich elements (AREs) at 3′ UTR to activate translation (55). Several miRNAs, including let-7, were found to be associated with AGO2 and FXR1 to activate translation during cell cycle arrest, but they inhibit translation in proliferating cells (55). Upregulation of gene expression by miRNAs was also observed in quiescent cells, such as oocytes (56, 57). The miRNA-mediated activation of translation involves AGO2 and FXR1 instead of GW182 (56). Other examples of gene activation by miRNAs include binding to the 5′ UTR of mRNAs encoding ribosomal proteins during amino acid starvation (58); thus suggesting that miRNA-mediated upregulation of gene expression occurs under specific conditions.

### MicroRNA-mediated transcriptional and post-transcriptional gene regulation within the nucleus

Through Importin-8 or Exportin-1, human AGO2 shuttles between the nucleus and cytoplasm via its interaction with TNRC6A (a GW182 family protein) which contains a nuclear localization and export signal (59). Nuclear localized miRISC was found to regulate both transcriptional rates and post-transcriptional levels of mRNA (59–61) and associate with euchromatin at gene loci with active transcription (62). However, our understanding of when and how miRNAs exert their functions in the nucleus is still limited.

It has been reported that low molecular weight miRISC can interact with mRNAs within the nucleus and induce nuclear mRNA degradation, although the mechanism behind this is unclear (59, 61, 63). Enrichment of miRNA at actively transcribed genes may suggest that miRISC interacts with target mRNA co-/post-transcriptionally. The involvement of AGO and Drosha in mRNA splicing (64, 65) further supports co-transcriptional miRISC:mRNA interactions. miRISC may also regulate transcription directly. A study showed that AGO2 was concentrated in the nucleus of senesced fibroblasts and interacted with miRISC and retinoblastoma (Rb) to suppress the transcription of proliferation-promoting genes regulated by Rb/E2F. It was noted that let-7f was bound to MREs found in the promoters of two E2F target genes, *CDCA8* and *CDC2*, in an AGO2-dependent manner (60). Also, a subset of AGO-promoter bound genes was upregulated following senescence, and AGO2 was found to co-immunoprecipitate with euchromatin (60). A more recent study by Miao et al. (66) found nuclear miR-522 interacting with a DNA cruciform structure (a stem-loop on sense and antisense DNA strands) within the promoter of *CYP2E1* and suppressing its transcription (66). miRNAs have also been shown to interact at genomic loci, where enhancer-derived RNA (eRNA) are transcribed, and increase mRNA levels of adjacent genes by promoting a transcriptionally active chromatin state (67) while altering alternative splicing profiles (64). The overall role of miRISC in the regulation of chromatin state and structure and transcriptional control remain to be determined, but these current data suggest a transcription factor-like role. It is also possible that miRISC may be involved in the establishment of *de novo* methylation, and by extension, the compactification of chromatin into nuclear compartments, and mediation of genomic remodeling.

## Dynamics of miRNA actions

Studies have revealed that miRNA-mediated gene regulation is dynamic and helps to buffer gene expression to a steady state. It is only recently that a more comprehensive understanding of miRNA dynamics has begun to shed light on the highly robust nature of miRNA-mediated gene regulation. Factors that may contribute to the robustness of miRNA-mediated gene regulation include the functionalized compartmentalization and shuttling of miRISC within the cells. The availability and abundancy of miRNAs and their target mRNAs are also contributing factors in determining which genes are regulated. Although this is not always the case, miRNA suppression of mRNA targets is not ubiquitous between cell types. Alternative splicing and alternative polyadenylation affecting 3′ UTRs, and cell type-specific RNA binding proteins that affect target mRNA secondary structures, change the available pool of MREs (63, 68, 69). This renders subsets of mRNAs sensitive or insensitive to miRNA-mediated gene regulation in a cell type/state-specific manner.

### Subcellular compartmentalization of miRNAs

**Figure 2:**
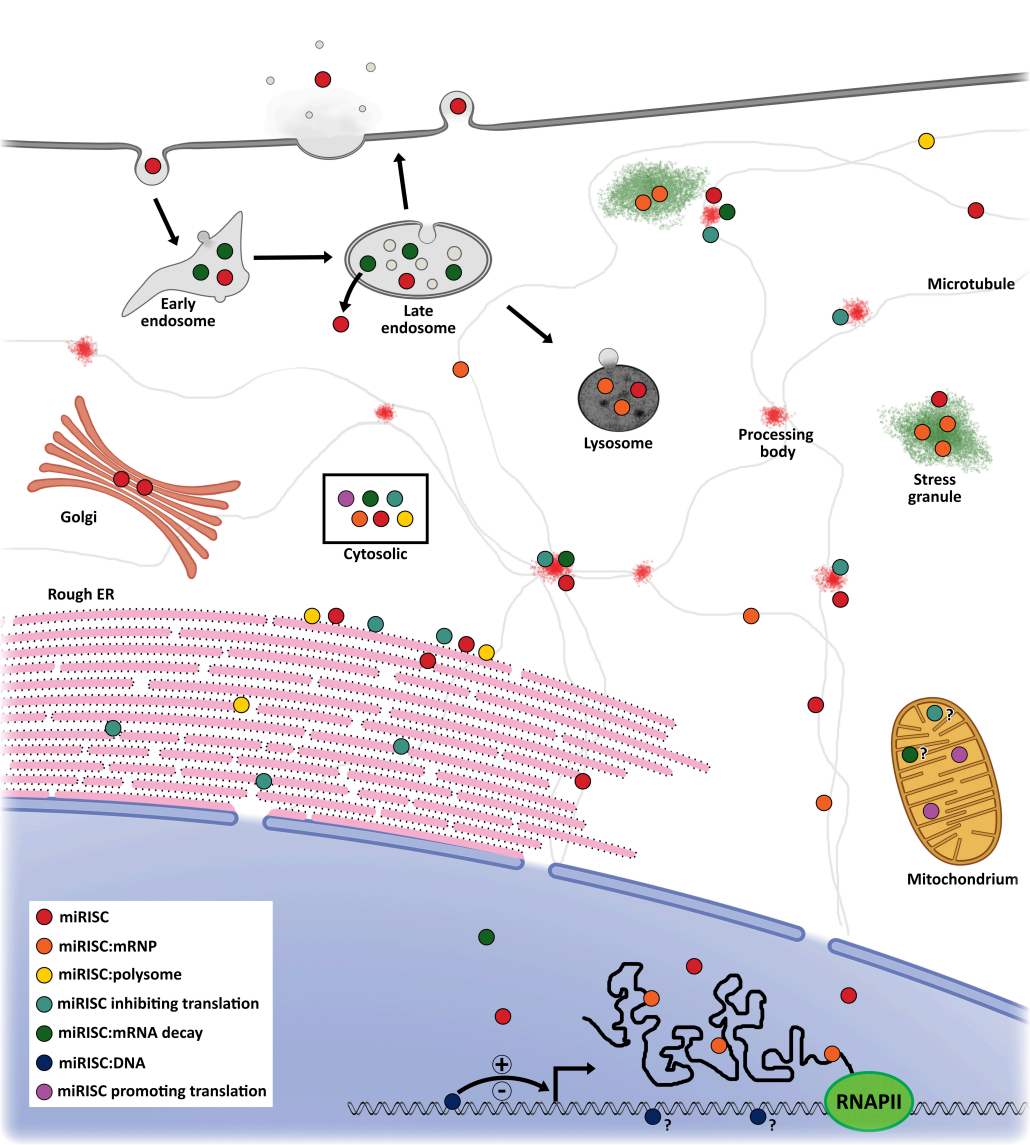
Proposed model of miRNA localization and function. miRISC has been detected in several subcellular locations. In the nucleus, miRISC is enriched at sites of active transcription where it can interact with DNA to promote active or inactive chromatin states. It can also interact with nascent mRNA to promote more efficient splicing or alternate splicing profiles. miRISC can interact with nuclear messenger ribonucleoprotein (mRNP) to promote its degradation or remain in A miRISC:mRNP complex as it is shuttled out of the nucleus. Cytoplasmic miRISC can diffuse throughout the cytosol or undergo shuttling, most likely via microtubules. Within the cytosol, miRISC can associate with polysomes, inhibit translation initiation, mediate mRNA decay, or promote translational activation. On the rough endoplasmic reticulum, miRISC can interact with translating mRNA to inhibit translation. Furthermore, unbound miRISC can also accumulate on the rER to interact with newly rER-bound mRNA. Rough ER miRISC:mRNP complexes that are translationally inhibited can shuttle to the early/late endosomes to complete mRNA deadenylation and decay. miRISC can then be recycled into the cytosol or shuttled to the lysosome for degradation. miRISC may also localize to transient, membrane-free processing bodies where it can mediate target mRNA translational inhibition and storage or decay. Under certain cellular conditions, miRISC:mRNPs may be shuttled to stress granules for storage and/or degradation. miRISC can also localize to the mitochondria to promote translational activation or mRNA translational inhibition and decay. Localization of miRISC within the Golgi is likely from vesicles secreted from the early endosome. Moreover, endocytosed miRISC may be shuttled to the Golgi or recycled into the cytosol. Lastly, vesicular or vesicle-free miRISC can be exocytosed from at least the late endosome into the extracellular milieu to mediate cell-cell communication.

miRISC and target mRNA have been observed to localize in multiple subcellular compartments including rough endoplasmic reticulum (rER) (70), processing (P)-bodies (71), stress granules (SG) (72), the trans-Golgi network (TGN), early/late endosomes (73), multivesicular bodies (MVB) (74), lysosomes (74), mitochondria (75, 76), and the nucleus (66, 77) (Figure 2). With regards to miRNA activity, the result is that components that facilitate miRNA post-transcriptional gene regulation are enriched at sites where miRISC:mRNA complexes localize. This in effect acts to spatially enrich mRNA and miRISC concentrations over time and promote efficient regulation of gene expression (70).

P-bodies were identified early on as possible sites involved with miRNA-mediated suppressive activity (78). They are cytoplasmic foci depleted of ribosomes (79) and enriched with enzymatic mRNA degradation machinery, such as mRNA decapping proteins, the CCR4-NOT complex, XRN1, and GW182 family proteins (78). Furthermore, P-bodies form in an RNA-dependent manner as a consequence of RNAi and RNA degradation (80). Recently, P-bodies with active miRISC have been associated with SYNE1 (nesprin-1) which acts to adhere Argonaute and other P-body components to microtubules (81). Disruption of nesprin-1 activity through mutations and RNAi significantly decreased miRNA suppression of target mRNA and P-body formation. On the other hand, stabilization of microtubules with Taxol led to translation inhibition but not mRNA decay of *HIF1A* following accumulation in P-bodies (82). Importantly, P-body accumulation was miRISC-dependent and reversible as recovery after Taxol treatment led to P-body dissociation back to control levels and *HIF1A* polysome re-association. It is not clear why a microtubule stabilization would lead to an increase in P-body count. The reduced polysome occupancy of *HIF1A* observed by Carbonaro et al. (82) may lead to a buildup of *HIF1A* mRNA on the static microtubule network promoting an increase in *HIF1A*-bound miRISC. This in turn may enrich miRISC on the static microtubule network and nucleate P-body formation. Typical microtubule instability may in effect dilute microtubule-associated miRISC by preventing enrichment at sites of nucleation. However, the degree to which P-bodies are necessary for efficient miRNA-mediated suppression is uncertain as mRNA degradation machinery exists diffusely throughout the cytoplasm, and at other subcellular locations, and can confer RNAi activities even in the absence of P-bodies (78).

Polysomes, which are complexes of mRNA with multiple translating ribosomes, are generally found freely within the cytoplasm or bound to the cytoskeleton or membranous subcellular organelles, such as rER. miRISC has also been found to copurify with polysomes, and the miRISC:mRNA complexes bound to these polysomes were associated with increased levels of translational inhibition and mRNA degradation (83). A recent study showed that miRNAs, AGO2, and target mRNAs are associated with rER-membrane-bound polysomes, and this occurs before miRNA-mediated translational repression (70). Further work by the same group has led to the finding that rER-bound mRNA, destined for miRNA-dependent degradation, complete this process within endosomes and MVBs followed by miRISC recycling back into the cytoplasm (73). Importantly, this interaction is miRNA-dependent; disruption of translation, micrococcal nuclease degradation of cytoplasmic mRNA, or mRNA mutants lacking cognate MRE led to a loss of polysome/miRISC copurification (70, 84). Additionally, the degree of polysome occupancy of target mRNAs is dependent on the affinity between miRNA seed region and MRE, but not on miRNA abundance (84). Moreover, at any given time the average proportion of polysome-bound and -unbound miRISC, called the polysome occupancy, was below 40%. These data are in agreement with high rates of miRNA-bound AGO sampling of targets observed *in vitro* (85), i.e., miRISC is able to probe for MREs quickly, but does not form stable interactions on its own. Together these data elude to a key aspect of miRNA functioning; there is a crucial equilibrium between MRE bound and unbound states and this equilibrium is important for the spatiotemporal dynamics of miRISC. This allows for miRNA to respond quickly to changes in subcellular environments and dynamically regulate many target mRNAs.

### A global view of miRNA at the cellular level

The interplay between miRNA abundance/localization, cell type, cell state, and miRNA-mediated regulation is still under intense investigation. Recently, the work by the Functional Annotation of the Mammalian Genome (FANTOM5) consortium found that for any given human cell type, the top five expressed miRNAs represent on average ~50% of the overall miRNA pool (13). Moreover, approximately half of the expressed miRNAs are cell type enriched, one quarter are broadly expressed, and the remaining had low-level expression regardless of cell type. These data help fortify the general roles that miRNA play within cells. Generally, miRNA can regulate gene networks dynamically and/or transiently, e.g., feedback and feedforward loops (86), and steady state gene regulation, e.g., upregulation of a miRNA throughout differentiation (87). In both cases, higher-order effects on gene regulatory networks can propagate (88), such as regulation of transcription factor expression leading to changes in transcriptional profiles. Intriguingly, miRNA do not solely, or maybe even predominantly, function as target-specific regulators but may play key roles in the post-transcriptional reduction of expression noise (89, 90). In this way miRNA promote stable gene expression by buffering out stochastic fluctuations in transcription. A common indicator of expression noise control is high mRNA to protein ratios, rendering reduced translational rates resistant to random fluctuations in mRNA concentration (91), i.e., a small change to a highly abundant mRNA has little effect on protein output. This behavior is seen throughout the animal and bacterial kingdoms, selecting for controlled protein output rather than coupling of mRNA/protein levels (92). Accordingly, the strongest predictor of protein level for any given gene is the rate of translation, followed by mRNA levels (93).

An important concept in connecting miRNA dynamics and cellular gene expression networks is the idea of MRE load (94). There is an MRE load for each miRNA which represents the total number of available binding sites on all targetable RNA molecules within a cell. Simply put, a miRNA may interact with or sample all available MREs, but the retention time will be longer for higher affinity MREs compared to lower affinity MREs (94). This has the effect of diluting cellular miRNAs amongst many potential targets such that only a small proportion of each target mRNA is bound to a cognate miRNA at any given time (94). On the other hand, mRNAs with higher affinity MREs generally show greater sensitivity to miRNA-mediated post-transcriptional repression (84, 94). Nevertheless, the balance between MRE load and cognate miRNA abundance can lead to varying effects. Increasing the MRE load of a single miRNA, by upregulating one gene, has the effect of sequestering that miRNA away from other targets (94, 95). In this way, modulation of MRE load with competing endogenous RNAs (ceRNAs), such as with circular RNAs (circRNAs) (96), can act to sequester miRISC from target mRNA, leading to derepression of target mRNA (97). To complicate matters further, by altering subcellular localizations of miRISC, the MRE load can be modulated. At sites of synaptic formation in neurons, rapid and highly specific RNAi takes place due to the colocalization of target mRNAs and cognate miRNAs (98).

An individual mRNA may contain many MREs (99), and thus contributes to multiple MRE loads for different miRNA. Although to some degree, each individual mRNA with multiple MREs may act as a ceRNA, multiple MREs can also act to increase miRISC occupancy. Cooperation of multiple, proximal MREs on target mRNA has been shown to increase RNAi activity and increase retention times of miRISC complexes on those mRNA, most likely through the three AGO binding sites on the GW182 family proteins (94, 100). In this way, low expression but functional miRNA may synergistically increase RNAi activity on a subset of target mRNAs, thereby effectively enhancing the concentration of cognate miRNAs for specific mRNAs (101, 102). Inversely, low miRNA levels can be compensated for, when target mRNA levels are high, by stimulating increased loading of cognate miRNA into AGO, thereby improving RNAi activity (103). Synergistic effects of miRNAs have been shown to be important for biological processes, such as neurogenesis (104) and human embryonic stem cell pluripotency and differentiation (105). Highly synergistic miRNA regulation of target mRNA may also have implications in diseases, such as oncogenesis. For example, cyclin-dependent kinase inhibitor 1A (CDKN1A), a tumor suppressor that is downregulated in multiple cancers (106), is targeted by at least 28 miRNAs and many of which are upregulated together in cancers where CDKN1A has been implicated (107). This may suggest that the upregulation of sets of miRNAs in these cancers can synergistically downregulate CDKN1A. miRNA synergism has also been exploited as a therapeutic strategy (108).

In addition to specific miRNA/mRNA dynamics, changes in global miRISC localization in response to changes in cellular environments, such as stress or serum starvation (109), also affect miRNA activity. In a study by Wang et al. (110), upon serum starvation of multiple human cell lines, there was an immediate exportation of stable, vesicular or vesicle-free miRNA and a concomitant decrease of intracellular miRNA levels (110). Both cell stress induced by heat shock and translation inhibition following treatment with hippuristanol or cycloheximide induced translocation of miRISC from the nucleus and cytoplasm to transient, cytoplasmic SG (72, 111). SG are known to act as intermediate storage of messenger ribonucleoproteins (mRNPs) that have stalled during translation or following viral infection (112). SG components can also return to the cytoplasm, exchange with P-bodies, or be digested by lysosomes (81, 112). miRISC that is localized to SG following cell stress also contained target mRNAs, leading to a reduction of RNAi activity. The extracellular release of miRNA upon cell stress and localization of miRNA:mRNA complexes to SG suggests an mRNA-protective role.

## Circulation of miRNAs

Numerous studies have demonstrated that miRNAs can be released into extracellular fluids. Extracellular miRNAs can be used as biomarkers for a variety of diseases. These studies have been extensively reviewed (113–115) and therefore will not be discussed here. The extracellular miRNAs can be delivered to target cells and they may act as autocrine, paracrine, and/or endocrine regulators to modulate cellular activities (116). In this regard, miRNAs have hormone-like activities.

### MicroRNAs in biological fluids

Many studies have detected extracellular/circulating miRNAs in biological fluids, such as plasma and serum (117, 118), cerebrospinal fluid (119), saliva (120), breast milk (121), urine, tears, colostrum, peritoneal fluid, bronchial lavage, seminal fluid (122), and ovarian follicular fluid (123). Contrary to cellular RNA species, extracellular miRNAs are highly stable, resisting degradation at room temperature for up to 4 days and in deleterious conditions such as boiling, multiple freeze-thaw cycles, and high or low pH (117, 124).

Two populations of extracellular miRNAs exist in biological fluids. One can be found in vesicles such as exosomes, microvesicles, and apoptotic bodies (116, 120) while the other is associated with proteins, especially AGO2 (120, 125). There have been some discrepancies on the relative abundancies of these two populations. While several studies found that the majority of extracellular miRNAs are not associated with exosomes/microvesicles but are instead bound to AGO2 (118, 125), another study reported that extracellular miRNAs are present predominantly in exosomes in human serum and saliva (120). Since these studies only measured a selected group of miRNAs in a few plasma samples, it is possible that the existence of predominantly exosomal or vesicle-free miRNAs is dependent on the miRNA itself, the cell type they originate from, and/or other factors affecting the secretion of miRNAs in individuals. Other proteins found to bind extracellular miRNAs include high-density lipoprotein (HDL) (126, 127) and nucleophosmin 1 (NPM1) (110, 118, 128). The presence of miRNAs in vesicles or with accompanying proteins is generally thought to protect extracellular miRNAs and increase their stability in the extracellular milieu (120).

### Secretion and uptake of microRNAs

Although some extracellular miRNAs are regarded as by-products of cellular activities, such as cell injury or death (128), increasing evidence suggests that the release of extracellular miRNAs is a regulated process. It has been shown that the secretion of exosomal miRNAs is mediated by a ceramide-dependent pathway and the secreted miRNAs exert growth regulatory effects in target cells (129). Recently, it was demonstrated that atheroprotective laminar shear stress induced the release of vesicle-free miR-126-3p and other miRNAs, as well as AGO2, from endothelial cells by activating vesicle-associated membrane protein 3 (VAMP3) and synaptosomal-associated protein 23 (SNAP23) (130). This study also showed that miRNAs secreted from endothelium can regulate the activity of smooth muscle cells (130). In neuroendocrine cells, miRNAs in large dense-core vesicles (LDCVs) are released by exocytosis through vesicle fusion, and this process is mediated by the SNARE complex and accelerated by Ca^2+^ stimulus (131). Secretion of miRNAs via exosomes have also been reported to be regulated by signaling molecules, such as interleukin-4 (IL4) (132) and Docosahexaenoic acid (DHA) (133). IL4-activated macrophages were found to secrete exosomes carrying oncogenic miRNAs to promote invasiveness of breast cancer cells (132). On the other hand, DHA, which has anticancer and anti-angiogenic activities, induced the secretion of miRNA-containing exosomes that exert inhibitory effects on tumor angiogenesis (133).

Many studies have also demonstrated that extracellular miRNAs can exert biological functions in recipient cells to regulate their activity, thereby acting as intercellular signaling molecules. For example, exosome mediated transfer of miR-105 from metastatic breast cancer cells to endothelial cells directly targeted a tight junction protein, zonula occludens 1 (ZO-1), and this led to the destruction of the barrier function of endothelium and promoted metastasis (134). Moreover, exosomes from umbilical cord blood were found to be enriched in miR-21-3p, which promoted the proliferation and migration of fibroblasts, and induced the angiogenic activities of endothelial cells, leading to accelerated wound healing (135). miRNAs, specifically miR-342–3p and miR-1246, secreted from a highly metastatic human oral cancer cell line, were found to induce metastasis in a poorly metastatic cancer cell line (136). Extracellular miRNAs have also been reported to bind to Toll-like receptors (137), activate downstream signaling events, and eventually lead to biological responses, such as tumor growth and metastasis (138), and neurodegeneration (139). Thus, miRNAs may act as chemical messengers to regulate cell-cell communications.

The mechanisms of extracellular miRNA uptake are not well understood. It has been proposed that vesicle-associated extracellular miRNAs may enter cells by endocytosis, phagocytosis, or direct fusion with the plasma membranes, while vesicle-free secreted miRNAs may be taken up by specific receptors on the cell surface (140). Indeed, several studies have shown that miRNAs enter recipient cells by endocytosis and micropinocytosis (141, 142). This process has been reported to be dependent on clathrin, but not on caveolae or lipid rafts in PC12 cells (142). However, another study conducted in A549-P cells showed that endocytosis of exosomal miRNAs is mediated by caveolae- and lipid raft-dependent pathways (143). Furthermore, vesicle-free miRNAs associated with HDL are taken up by HDL receptor and scavenger receptor BI (SR-BI) receptor in the plasma membrane of the target cells (126, 127). miRNAs have also been shown to transfer between co-cultured cells via direct cell-cell contact and gap junctions (144). While these studies suggest that extracellular miRNAs can interact with recipient cells via multiple mechanisms, the factors that determine the specificity of such interactions need to be investigated.

## Concluding remarks

Since the discovery of miRNAs in the earlier 1990s, tremendous progress has been made on how miRNAs are produced within cells, how they exert regulatory effects on gene expression, and how they are involved in various physiological and pathological events. It is now clear that miRNAs are powerful gene regulators, and that they not only help control mRNA stability and translation but are also involved in transcription. However, our understanding of when and how miRNAs can exert regulatory effects on transcription is limited. Similarly, the conditions under which miRNAs elicit translational activation need to be further explored. In addition, careful analysis and consideration of experimental techniques and model systems should be employed when attempting to generalize miRNA capabilities. The assaying of miRNA activity within a test tube may not be recapitulated within the cellular environment and thus should be viewed with caution. Many studies have been conducted *in vitro* by transfecting pre-miRNAs or mature mRNA mimics into immortalized and cancer cell lines. The extent to which findings from such studies reflect the endogenous miRNA functions *in vivo* requires further study. Also, the addition of chemical tags to miRNA could also impact miRNA:MRE interaction or AGO:miRNA interactions, specifically with 5′ and 3′ miRNA nucleotide modifications. The first and last miRNA nucleotides clamp the miRNA within AGO proteins, and thus tags to these regions may affect miRNA functioning in unpredictable ways.

Recent studies have shed light on the dynamic nature of miRNA actions and further revealed the complexity of miRNA-mediated gene regulation. Many factors contribute to the activity of miRNAs, including subcellular location, miRNA/mRNA abundance, miRNA:MRE affinity, cell type/state, and the availability of various miRISC components. miRNAs in the nucleus play a role in regulating transcription and alternative splicing. Cytosolic miRISC components shuttle between different compartments. Moreover, their localization, together with the levels of miRNAs and target mRNAs, and the affinity of the miRNA-mRNA interaction, are important for efficient gene regulation. Recent advances in single molecule imaging will greatly impact the field, as has already begun. Viewing the movement of single miRNA and/or mRNA with high spatial and temporal resolution will help us understand this complex dynamic in an unprecedented way. Investigation of large scale, global miRNA interactomes will also propel the field forward, allowing powerful mathematical models to be applied to highly complex regulatory networks.

It is now accepted that extracellular/circulating miRNAs can not only serve as biomarkers for diseases, but also play important roles in intercellular communication. miRNAs regulate the activity of host cells, and they are also secreted and transferred to recipient cells. Many studies have shown that extracellular miRNAs are functionally active in recipient cells. Some miRNAs can even interact with cell surface receptors, such as Toll-like receptors. Therefore, miRNAs have hormone-like activities. However, most studies conducted so far were done *in vitro* using co-culture of different cell types. More *in vivo* studies are required to determine whether miRNAs target specific cells under physiological conditions. Although miRceptors have been proposed (137), apart from the Toll-like receptors, they remain to be identified. Mechanisms by which miRNAs are secreted and taken up by cells are not well understood and require further investigation.

## Author contributions

All authors listed have made a substantial, direct and intellectual contribution to the work, and approved it for publication.

### Conflict of interest statement

The authors declare that the research was conducted in the absence of any commercial or financial relationships that could be construed as a potential conflict of interest.
